# How do serum cytokine levels change in myocarditis and inflammatory dilated cardiomyopathy relative to healthy individuals? A protocol for a systematic review and meta-analysis

**DOI:** 10.1136/bmjopen-2024-087052

**Published:** 2025-02-07

**Authors:** Piotr Lewandowski, Maciej Baron, Marcin A Rojek, Marcin Goławski, Karol Krystek, Magdalena Żegleń, Pascal Zytkiewicz, Jakub Kancerek, Klaudia Gabriela Lis, Jakub Warecki, Jakub Kufel, Kamila Kuśpiel, Romuald Wojnicz

**Affiliations:** 1 Department of Histology and Cell Pathology, Medical University of Silesia, Zabrze, Poland; 2 Department of Pharmacology, Medical University of Silesia, Zabrze, Poland; 3 Department of Radiodiagnostics, Invasive Radiology and Nuclear Medicine, Department of Radiology and Nuclear Medicine, Medical University of Silesia, Zabrze, Poland; 4 Silesian Nanomicroscopy Center, Medical University of Silesia, Zabrze, Poland

**Keywords:** Cardiomyopathy, CARDIOLOGY, IMMUNOLOGY, Systematic Review, Meta-Analysis

## Abstract

**Abstract:**

**Introduction:**

Myocarditis is an inflammatory heart disease resulting from infections, toxic exposures or autoimmune reactions. Irrespective of the factors responsible for this disease, cytokines play an important role in regulating the immunological response involved in its development and progression. Accordingly, this protocol aims to conduct a systematic review and meta-analysis summarising previous research on serum and plasma levels of cytokines in patients with myocarditis and inflammatory dilated cardiomyopathy.

**Methods and analysis:**

Four scientific databases: PubMed, Embase, Scopus and Web of Science, will be searched. The estimated date of the search will be 30 March 2024. Each stage of the review, including the study selection, data extraction, risk of bias and quality of evidence assessments, will be performed in duplicate. Studies meeting the following criteria will be eligible for inclusion: (1) studies involving ‘myocarditis’ or ‘inflammatory dilated cardiomyopathy’ and (2) studies are required to report serum levels of any cytokine. Meta-analyses will be used to summarise serum levels of each cytokine if possible. Subgroup analysis will be stratified by age, sex, sample size, New York Heart Association scale, cardiac Troponin T, N-terminal prohormone of brain natriuretic peptide, C reactive protein, number of lymphocytes per mm^2^ in the endomyocardial biopsy.

**Ethics approval and dissemination:**

This study does not require ethics approval. After completion, the results will be published in a peer-reviewed paper. Data generated during the study will be published in an open access repository.

**PROSPERO registration number:**

CRD42024519625

**STRENGTHS AND LIMITATIONS OF THIS STUDY**

- This study will follow Preferred Reporting Items for Systematic Reviews and Meta-Analyses guidelines for reporting protocols guidelines to ensure rigorous and transparent reporting of the systematic review protocol.
- A comprehensive risk of bias assessment and certainty of evidence evaluation will be conducted for all included studies.
- A meta-analysis approach will be used to synthesise quantitative data and identify patterns in cytokine levels.
- The main limitation of this study could be the high heterogeneity of included studies and patient cohorts, and this may lead to inconsistent results.

## Background

### Rationale for this study

 Myocarditis (MCI) is an inflammatory heart disease that occurs as a consequence of infections, exposure to toxic substances and autoimmune reactions.^1^,^3^ The Global Burden of Disease Study estimated the annual incidence of MCI to be 22 cases per 100 000 patients^4^. Furthermore, it is a relatively common cause of sudden cardiac death among young people (about 6% in autopsy-based series).^5^ Clinically, two subtypes of MCI are based on the symptom duration: acute and chronic. Acute MCI is recognised when symptoms occur rapidly, regularly leading to an early diagnosis (typically less than a month). Chronic MCI indicates myocardial inflammation with a longer duration of symptoms (usually defined as more than a month). Chronic MCI can lead to cardiac dysfunction and ventricular remodelling and may transition into inflammatory dilated cardiomyopathy (iDCM).^2 6^

The specific mechanism of pathogenesis of MCI and transition to iDCM is not fully understood. However, it is considered a multifactorial process, with several immunological mechanisms contributing to the disease development and progression. It is believed that auto-inflammatory reactions might have a crucial role in this disease. Inflammatory cells infiltrate cardiac tissue leading to heart damage and remodelling. One of the key factors regulating this process is cytokines.^7^

Cytokines are low molecular weight (mainly~40–80 kD) soluble proteins, secreted by virtually all nucleated cells, although they are typically associated with the immune cells (lymphocytes, macrophages, mast cells, stromal cells, etc). They engage in the inflammation as a danger signal and also as a communication link between the immunocompetent cells, overseeing the process. However, abnormalities in the production of pro-inflammatory cytokine storms are observed in various pathologies including MCI and iDCM.^8 9^ The role of cytokines in the aforementioned diseases is still an object of intensive research. Some scientific evidence suggests their potential significance in the aetiology of MCI and iDCM. For example, interleukin (IL)-1 exerts pro-apoptotic and hypertrophic effects on cardiomyocytes, depressing cardiac contractility^10^ and IL-6 overexpression correlates with a worse prognosis in patients with MCI.^11^

Interestingly, the usefulness of cytokine inhibitors in MCI and iDCM is currently being investigated. An example of such an inhibitor is Anakinra, a recombinant human IL-1 receptor antagonist, which may effectively treat pericarditis.^12 13^ In the paediatric population, it was observed that anakinra improves the myocardial function of patients with fulminant MCI.^13^ On the other hand, the ARAMIS (Anakinra vs. placebo double-blind, Randomized controlled trial for the treatment of Acute MyocarditIS’) study results are less optimistic, revealing that there is no notable improvement in the condition of patients with MCI.^14 15^

### Study objectives

The aim of this systematic review and meta-analysis is to summarise previous research on serum and plasma levels of cytokines in patients with myocarditis and inflammatory dilated cardiomyopathy. To this end, the proposed study will answer the following questions:

1. Which cytokines have already been evaluated in acute/chronic myocarditis and inflammatory dilated cardiomyopathy patients?
2. Do the serum/plasma concentrations in myocarditis and inflammatory dilated cardiomyopathy patients differ compared with healthy volunteers?
3. Do serum/plasma cytokine concentrations differ between chronic myocarditis/inflammatory dilated cardiomyopathy and acute myocarditis patients?
4. What is the relationship between cytokine concentrations and prognosis of myocarditis?
5. What clinical and methodological characteristics explain the heterogeneity in results (if heterogeneity is present)?

## Methods and analysis

This protocol for the systematic review and meta-analysis was registered in the PROSPERO International Prospective Register of Systematic Reviews. The methods for conducting and describing the results of this systematic review and meta-analysis will be reported in accordance with the Preferred Reporting Items for Systematic Reviews and Meta-Analyses 2020 (PRISMA 2020).^16^ The protocol has been prepared according to PRISMA guidelines for reporting protocols (PRISMA-P.^17 18^ The PRISMA-P checklist is available as online supplemental material S1. This systematic review and meta-analysis was started in March 2024. The estimated completion date is June 2025. The protocol has been registered in PROSPERO database (CRD42024519625)^19^ .

### Eligibility criteria

#### Study designs and participants

We will include prospective and retrospective observational studies and clinical trials (baseline data only). We will exclude case series and case reports. Studies examining the general population will be included as a comparator.

#### Clinical diagnosis

We will include studies addressing myocarditis and inflammatory dilated cardiomyopathy. All studies describing ‘myocarditis’ or ‘inflammatory dilated cardiomyopathy’ will be included. Studies reporting ‘dilated cardiomyopathy’ or ‘idiopathic dilated cardiomyopathy’ will be included only if:

1. Inflammation was not excluded by endomyocardial biopsy;
2. Coronary artery disease was excluded; and
3. Authors did not identify other causes of DCM.

Studies involving the following diagnoses will be included: ‘active myocarditis’, ‘acute myocarditis’, ‘chronic inflammatory cardiomyopathy’, ‘chronic myocarditis’ and ‘fulminant myocarditis’. The exclusion criteria involve the following diagnoses: ‘sarcoidosis’, ‘amyloidosis’, ‘eosinophilic myocarditis’, ‘pericarditis’, ‘drug-induced myocarditis’ and ‘diabetic cardiomyopathy’. Additionally, myocarditis caused by parasites (ie, *Trypanosoma cruzi*) will be excluded. Study groups involving conditions/diseases leading to myocarditis or known for abnormal cytokine expression will also be excluded, that is, heart transplant recipients, rheumatic diseases, COVID-19 and sepsis. Healthy volunteers will be included as controls. Included studies may or may not have a comparator group.

#### Outcomes

Eligible studies are required to report serum levels of any cytokine. The following proteins will be eligible as ‘cytokines’: (1) interleukins, (2) interferons, (3) tumour necrosis factor alpha and beta and (4) chemokines. Only direct protein level measurements will be valid. Measurements of mRNA levels or flow cytometry measurements of cells expressing cytokines will be excluded. Additionally, the following outcomes will be recorded (if available): New York Heart Association (NYHA) functional class, ejection fraction (EF), number of lymphocytes per mm^2^ in an endomyocardial biopsy and serum levels of C reactive protein (CRP), N-terminal prohormone of brain natriuretic peptide (NT-proBNP), cardiac troponin T(cTnT).

### Information sources and search strategy

A literature search strategy will be performed using a prepared search key using medical subjects’ headings (MeSH)/Embase subject headings (Emtree) and text words related to myocarditis/dilated cardiomyopathy and cytokines. We will search four scientific databases: PubMed, Embase, Scopus and Web of Science. The search will be performed by two authors (MAR and MB) with any disagreements resolved by discussion with the third author (PL). The search will not be limited by date or language of publication using search syntax presented in online supplemental material S2. All search results will be uploaded into a Microsoft Excel worksheet.

### Data management

One researcher (MAR) will be responsible for data management. Records from all searches will be merged into one worksheet and each record will get a unique ID. Duplicates will be removed using the manual gold standard method described by Kwon *et al*.^20^ The data manager will prepare files for the title and abstract screening, full-text screening, blinding and maintaining data integrity. All full texts of included records will be uploaded into the OneDrive folder using a predefined filename format: surname_year-of-publication.pdf. In the event of protocol amendments, the date of each amendment will be accompanied by a description of the change and the rationale.

### Selection of studies

Seven authors (MB, MZ, KK, PZ, JK, KL and JW) working as pairs of reviewers will independently screen the titles and abstracts of the retrieved articles. Then, the full texts of potentially eligible records will be independently screened for eligibility by two reviewers (PL and MG). Any discrepancies during these steps will be resolved by a discussion with another author (RW). The results of the study selection, together with the reasons for full-text article exclusion, will be presented using a PRISMA-compliant flow diagram. If more than one report from the study is available, all of them will be included unless data are redundant or discrepant.

### Data extraction

Data extraction will be performed using a standardised Excel worksheet to extract data for synthesis and risk of bias assessment. Seven authors (MB, MZ, KK, PZ, JK, KL and JW) working as pairs of reviewers will independently extract data from each record. Any differences will be resolved by two reviewers (PL and MG). To ensure consistency across reviewers, we will conduct calibration exercises before starting the review. The following data are planned to be extracted: first author, year of publication, country, patients demographic characteristics (age, sex, ethnicity), diagnosis, methodology of confirmation (ie, cardiac MRI, endomyocardial biopsy), diagnosis criteria (ie, Dallas criteria, ESC 2021), heart failure and general biomarkers (NYHA, EF, cTNT, NT-proBNP, CRP, number of lymphocytes per mm^2^ in endomyocardial biopsy), cytokine concentrations and methods of measurement. Additionally, if possible, we will extract data on the relationship between cytokine concentration and patient prognosis. Such data will include, for example: (1) HRs for death with CI per unit of cytokine concentration, (2) cytokine concentrations and their respective SD in groups that survived and in groups that died during the follow-up and (3) diagnostic OR, sensitivity, and specificity of cytokine testing in myocarditis for death prediction.

### Risk of bias assessment

To facilitate the assessment of the risk of bias within included studies, each record will be evaluated using the Effective Public Health Practice Project (EPHPP)^21^ tool by two independent reviewers (MB and KK). Any discrepancies during these steps will be resolved by a discussion with another author (PL). To ensure consistency and reproducibility of each assessment, an Excel worksheet and criteria explanation manual will be prepared.

### Data synthesis

We will conduct meta-analyses for all cytokines measured in at least two studies. The weighted mean serum concentration will be calculated for each cytokine for the case and control group. For each record, the standard mean difference and 95% CI of cytokine concentration between case and control group will be calculated. In the case of studies without a control group, the mean difference will be calculated based on the weighted mean of all control groups in the included studies. The percentage variability across studies will be calculated using I^2^ statistic (0–40%: might not be important; 30–60%: may represent moderate heterogeneity; 50–90%: may represent substantial heterogeneity; 75–100%: considerable heterogeneity). If high levels of heterogeneity among the trials exist (I^2^>=50% or p <0.1) study design and characteristics of the included studies will be analysed. We will try to explain the source of heterogeneity by subgroup analysis or sensitivity analysis. If the I^2^ value is more than 50%, the analysis will be performed using a random-effect model; otherwise, a fixed-effect model will be used. To explore potential sources of inconsistency and heterogeneity, we will conduct subgroup and sensitivity analysis. Subgroup analysis will be stratified by variables such as age, sex, sample size, NYHA scale, cTNT, NT-proBNP, CRP and number of lymphocytes per mm^2^ in the endomyocardial biopsy. The sensitivity analyses will include (1) studies with myocarditis confirmed by endomyocardial biopsy and (2) studies with low and moderate risk of bias. A systematic narrative synthesis will be provided with the information presented in the text and tables to summarise and explain the characteristics and findings of the included studies. Additionally, the potential for reporting bias will be explored by funnel plots if ≥10 studies are available. All analyses will be performed in Python and the PythonMeta package.

### Certainty of evidence assessment

We will evaluate the credibility of the evidence for all outcomes using the methodology established by the Grading of Recommendations Assessment, Development and Evaluation working group. We will scrutinise the quality of evidence across various dimensions including susceptibility to bias, coherence, relevance, accuracy and potential publication biases. Additional aspects may be considered as deemed appropriate. The evaluation will categorise the quality as either high (unlikely that additional research will significantly alter our confidence in the effect estimate), moderate (further research could substantially influence our confidence in the effect estimate and might lead to a different estimate), low (additional research is highly likely to impact our confidence in the effect estimate and could result in a different estimate) or very low (considerable uncertainty surrounding the effect estimate).

## Discussion

The preliminary searches have shown that cytokine serum levels have been extensively researched in patients with MCI and iDCM. Studies have demonstrated that certain cytokines are implicated in the pathogenesis of MCI or correlated with clinical outcomes. Clinical trials investigating the utilisation of IL-1β inhibitors in myocarditis treatment are currently undergoing. Early results indicate that cytokines may play a significant role in the development of novel diagnostic and therapeutic strategies for MCI and iDCM.

The upcoming systematic review and meta-analysis will represent the first attempt to consolidate and analyse data on cytokine levels in the serum of patients with MCI and iDCM. To ensure the provision of relevant and high-quality data, the meta-analysis will adhere to the PROSPERO guidelines. Given the diverse range of study types, including randomised controlled trials, case–control studies and retrospective studies, we have chosen to evaluate the risk of bias using the EPHPP scale, as recommended by Mamikutty *et al*, which is suitable for multidesign studies.^22^ Additionally, considering the anticipated high heterogeneity among study groups, we plan to conduct further subgroup analyses.^22^

Our findings will enhance and consolidate the existing knowledge regarding cytokine levels in MCI and iDCM. These results may resolve the cause of observed diversity in MCI and iDCM trials conducted across various countries and time periods. Moreover, our results will help identify the gaps in research and limitations of previous trials, offering insights that may be addressed in future studies.

## Ethics approval and dissemination

This study does not require ethics approval. After completion results will be published as a peer reviewed paper. Data generated during the study will be published in an open access repository.

## supplementary material

10.1136/bmjopen-2024-087052 online supplemental file 1

10.1136/bmjopen-2024-087052 online supplemental file 2
