# Supplementary material for: How do serum cytokine levels change in myocarditis and inflammatory dilated cardiomyopathy relative to healthy individuals? A protocol for a systematic review and meta-analysis
Source: BMJ Open. 2025 Feb 7;15(2):e087052. doi: 10.1136/bmjopen-2024-087052 (PMC11808896; doi:10.1136/bmjopen-2024-087052)
Supplement: online supplemental file 2 [file bmjopen-15-2-s002.docx]

**Supplementary materials S2 – search syntax**

PubMed

("Myocarditis"[Mesh] OR myocarditis OR "Cardiomyopathy, Dilated"[Mesh] OR dilated cardiomyopathy OR dcm) AND ("Cytokines"[Mesh] OR cytokine* OR interleukin* OR chemokine* OR “chemokines”[Mesh] OR “Tumor Necrosis Factors”[Mesh] OR “Interferons”[Mesh] OR il OR il1 OR il1a OR il1b OR il2 OR il3 OR il4 OR il5 OR il6 OR il7 OR il8 OR il9 OR il10 OR il11 OR il12 OR il13 OR il14 OR il15 OR il16 OR il17 OR il18 OR il19 OR il20 OR il21 OR il22 OR il23 OR il24 OR il25 OR il26 OR il27 OR il28 OR il28* OR il29 OR il30 OR il31 OR il32 OR il33 OR il35 OR il36 OR CCL1 OR CCL2 OR CCL3 OR CCL4 OR CCL5 OR CCL6 OR CCL7 OR CCL8 OR CCL9 OR CCL10 OR CCL11 OR CCL12 OR CCL13 OR CCL14 OR CCL15 OR CCL16 OR CCL17 OR CCL18 OR CCL19 OR CCL20 OR CCL21 OR CCL22 OR CCL23 OR CCL24 OR CCL25 OR CCL26 OR CCL27 OR CCL28 OR CXCL1 OR CXCL2 OR CXCL3 OR CXCL4 OR CXCL5 OR CXCL6 OR CXCL7 OR CXCL8 OR CXCL9 OR CXCL10 OR CXCL11 OR CXCL12 OR CXCL13 OR CXCL14 OR CXCL15 OR CXCL16 OR CXCL17 OR XCL1 OR XCL2 OR CX3CL1 OR tnf OR “tumour necrosis factor*” OR “tumor necrosis factor*” OR interferon* OR IFN OR ifnα OR ifnβ OR ifnε OR ifκ OR ifnτ OR ifnδ OR ifnζ OR ifnω OR ifnγ OR ifnλ*) AND (human* OR “humans”[Mesh] OR man OR men OR woman OR women OR person* OR “persons”[Mesh] OR people OR individual* OR patient* OR child* OR adolescent* OR newborn* OR toddler* OR senior*)

Embase

(‘myocarditis’/exp OR myocarditis OR ‘dilated cardiomyopathy’/exp OR dilated cardiomyopathy OR dcm) AND (‘cytokine’/exp OR cytokine* OR interleukin* OR chemokine* OR il OR il1 OR il1a OR il1b OR il2 OR il3 OR il4 OR il5 OR il6 OR il7 OR il8 OR il9 OR il10 OR il11 OR il12 OR il13 OR il14 OR il15 OR il16 OR il17 OR il18 OR il19 OR il20 OR il21 OR il22 OR il23 OR il24 OR il25 OR il26 OR il27 OR il28 OR il28* OR il29 OR il30 OR il31 OR il32 OR il33 OR il35 OR il36 OR CCL1 OR CCL2 OR CCL3 OR CCL4 OR CCL5 OR CCL6 OR CCL7 OR CCL8 OR CCL9 OR CCL10 OR CCL11 OR CCL12 OR CCL13 OR CCL14 OR CCL15 OR CCL16 OR CCL17 OR CCL18 OR CCL19 OR CCL20 OR CCL21 OR CCL22 OR CCL23 OR CCL24 OR CCL25 OR CCL26 OR CCL27 OR CCL28 OR CXCL1 OR CXCL2 OR CXCL3 OR CXCL4 OR CXCL5 OR CXCL6 OR CXCL7 OR CXCL8 OR CXCL9 OR CXCL10 OR CXCL11 OR CXCL12 OR CXCL13 OR CXCL14 OR CXCL15 OR CXCL16 OR CXCL17 OR XCL1 OR XCL2 OR CX3CL1 OR tnf* OR “tumour necrosis factor*” OR “tumor necrosis factor*” OR interferon* OR IFN*) AND (human* OR man OR men OR woman OR women OR person* OR people OR individual* OR patient* OR child* OR adolescent* OR newborn* OR toddler* OR senior*)

Scopus

(myocarditis OR (dilated W/2 cardiomyopathy) OR dcm) AND (cytokine* OR interleukin* OR chemokine* OR il OR il OR il1 OR il1a OR il1b OR il2 OR il3 OR il4 OR il5 OR il6 OR il7 OR il8 OR il9 OR il10 OR il11 OR il12 OR il13 OR il14 OR il15 OR il16 OR il17 OR il18 OR il19 OR il20 OR il21 OR il22 OR il23 OR il24 OR il25 OR il26 OR il27 OR il28 OR il28* OR il29 OR il30 OR il31 OR il32 OR il33 OR il35 OR il36 OR CCL1 OR CCL2 OR CCL3 OR CCL4 OR CCL5 OR CCL6 OR CCL7 OR CCL8 OR CCL9 OR CCL10 OR CCL11 OR CCL12 OR CCL13 OR CCL14 OR CCL15 OR CCL16 OR CCL17 OR CCL18 OR CCL19 OR CCL20 OR CCL21 OR CCL22 OR CCL23 OR CCL24 OR CCL25 OR CCL26 OR CCL27 OR CCL28 OR CXCL1 OR CXCL2 OR CXCL3 OR CXCL4 OR CXCL5 OR CXCL6 OR CXCL7 OR CXCL8 OR CXCL9 OR CXCL10 OR CXCL11 OR CXCL12 OR CXCL13 OR CXCL14 OR CXCL15 OR CXCL16 OR CXCL17 OR XCL1 OR XCL2 OR CX3CL1 OR tnf* OR “tumour necrosis factor*” OR “tumor necrosis factor*” OR interferon* OR IFN*) AND (human* OR man OR men OR woman OR women OR person* OR people OR individual* OR patient* OR child* OR adolescent* OR newborn* OR toddler* OR senior*)

Web of Science

(TS=(myocarditis OR (dilated NEAR/2 cardiomyopathy) OR dcm) OR TI=(myocarditis OR (dilated NEAR/2 cardiomyopathy) OR dcm) OR AB=(myocarditis OR (dilated NEAR/2 cardiomyopathy) OR dcm) OR AK=(myocarditis OR (dilated NEAR/2 cardiomyopathy) OR dcm) OR KP=(myocarditis OR (dilated NEAR/2 cardiomyopathy) OR dcm) OR SU=(myocarditis OR (dilated NEAR/2 cardiomyopathy) OR dcm) OR WC=(myocarditis OR (dilated NEAR/2 cardiomyopathy) OR dcm) ) AND (TS=(cytokine* OR interleukin* OR chemokine* OR il OR il1 OR il1a OR il1b OR il2 OR il3 OR il4 OR il5 OR il6 OR il7 OR il8 OR il9 OR il10 OR il11 OR il12 OR il13 OR il14 OR il15 OR il16 OR il17 OR il18 OR il19 OR il20 OR il21 OR il22 OR il23 OR il24 OR il25 OR il26 OR il27 OR il28 OR il28* OR il29 OR il30 OR il31 OR il32 OR il33 OR il35 OR il36 OR CCL1 OR CCL2 OR CCL3 OR CCL4 OR CCL5 OR CCL6 OR CCL7 OR CCL8 OR CCL9 OR CCL10 OR CCL11 OR CCL12 OR CCL13 OR CCL14 OR CCL15 OR CCL16 OR CCL17 OR CCL18 OR CCL19 OR CCL20 OR CCL21 OR CCL22 OR CCL23 OR CCL24 OR CCL25 OR CCL26 OR CCL27 OR CCL28 OR CXCL1 OR CXCL2 OR CXCL3 OR CXCL4 OR CXCL5 OR CXCL6 OR CXCL7 OR CXCL8 OR CXCL9 OR CXCL10 OR CXCL11 OR CXCL12 OR CXCL13 OR CXCL14 OR CXCL15 OR CXCL16 OR CXCL17 OR XCL1 OR XCL2 OR CX3CL1 OR tnf* OR “tumour necrosis factor*” OR “tumor necrosis factor*” OR interferon* OR IFN*) OR TI=(cytokine* OR interleukin* OR chemokine* OR il OR il1 OR il1a OR il1b OR il2 OR il3 OR il4 OR il5 OR il6 OR il7 OR il8 OR il9 OR il10 OR il11 OR il12 OR il13 OR il14 OR il15 OR il16 OR il17 OR il18 OR il19 OR il20 OR il21 OR il22 OR il23 OR il24 OR il25 OR il26 OR il27 OR il28 OR il28* OR il29 OR il30 OR il31 OR il32 OR il33 OR il35 OR il36 OR CCL1 OR CCL2 OR CCL3 OR CCL4 OR CCL5 OR CCL6 OR CCL7 OR CCL8 OR CCL9 OR CCL10 OR CCL11 OR CCL12 OR CCL13 OR CCL14 OR CCL15 OR CCL16 OR CCL17 OR CCL18 OR CCL19 OR CCL20 OR CCL21 OR CCL22 OR CCL23 OR CCL24 OR CCL25 OR CCL26 OR CCL27 OR CCL28 OR CXCL1 OR CXCL2 OR CXCL3 OR CXCL4 OR CXCL5 OR CXCL6 OR CXCL7 OR CXCL8 OR CXCL9 OR CXCL10 OR CXCL11 OR CXCL12 OR CXCL13 OR CXCL14 OR CXCL15 OR CXCL16 OR CXCL17 OR XCL1 OR XCL2 OR CX3CL1 OR tnf* OR “tumour necrosis factor*” OR “tumor necrosis factor*” OR interferon* OR IFN*) OR AB=(cytokine* OR interleukin* OR chemokine* OR il OR il1 OR il1a OR il1b OR il2 OR il3 OR il4 OR il5 OR il6 OR il7 OR il8 OR il9 OR il10 OR il11 OR il12 OR il13 OR il14 OR il15 OR il16 OR il17 OR il18 OR il19 OR il20 OR il21 OR il22 OR il23 OR il24 OR il25 OR il26 OR il27 OR il28 OR il28* OR il29 OR il30 OR il31 OR il32 OR il33 OR il35 OR il36 OR CCL1 OR CCL2 OR CCL3 OR CCL4 OR CCL5 OR CCL6 OR CCL7 OR CCL8 OR CCL9 OR CCL10 OR CCL11 OR CCL12 OR CCL13 OR CCL14 OR CCL15 OR CCL16 OR CCL17 OR CCL18 OR CCL19 OR CCL20 OR CCL21 OR CCL22 OR CCL23 OR CCL24 OR CCL25 OR CCL26 OR CCL27 OR CCL28 OR CXCL1 OR CXCL2 OR CXCL3 OR CXCL4 OR CXCL5 OR CXCL6 OR CXCL7 OR CXCL8 OR CXCL9 OR CXCL10 OR CXCL11 OR CXCL12 OR CXCL13 OR CXCL14 OR CXCL15 OR CXCL16 OR CXCL17 OR XCL1 OR XCL2 OR CX3CL1 OR tnf* OR “tumour necrosis factor*” OR “tumor necrosis factor*” OR interferon* OR IFN*) OR AK=(cytokine* OR interleukin* OR chemokine* OR il OR il1 OR il1a OR il1b OR il2 OR il3 OR il4 OR il5 OR il6 OR il7 OR il8 OR il9 OR il10 OR il11 OR il12 OR il13 OR il14 OR il15 OR il16 OR il17 OR il18 OR il19 OR il20 OR il21 OR il22 OR il23 OR il24 OR il25 OR il26 OR il27 OR il28 OR il28* OR il29 OR il30 OR il31 OR il32 OR il33 OR il35 OR il36 OR CCL1 OR CCL2 OR CCL3 OR CCL4 OR CCL5 OR CCL6 OR CCL7 OR CCL8 OR CCL9 OR CCL10 OR CCL11 OR CCL12 OR CCL13 OR CCL14 OR CCL15 OR CCL16 OR CCL17 OR CCL18 OR CCL19 OR CCL20 OR CCL21 OR CCL22 OR CCL23 OR CCL24 OR CCL25 OR CCL26 OR CCL27 OR CCL28 OR CXCL1 OR CXCL2 OR CXCL3 OR CXCL4 OR CXCL5 OR CXCL6 OR CXCL7 OR CXCL8 OR CXCL9 OR CXCL10 OR CXCL11 OR CXCL12 OR CXCL13 OR CXCL14 OR CXCL15 OR CXCL16 OR CXCL17 OR XCL1 OR XCL2 OR CX3CL1 OR tnf* OR “tumour necrosis factor*” OR “tumor necrosis factor*” OR interferon* OR IFN*) OR KP=(cytokine* OR interleukin* OR chemokine* OR il OR il1 OR il1a OR il1b OR il2 OR il3 OR il4 OR il5 OR il6 OR il7 OR il8 OR il9 OR il10 OR il11 OR il12 OR il13 OR il14 OR il15 OR il16 OR il17 OR il18 OR il19 OR il20 OR il21 OR il22 OR il23 OR il24 OR il25 OR il26 OR il27 OR il28 OR il28* OR il29 OR il30 OR il31 OR il32 OR il33 OR il35 OR il36 OR CCL1 OR CCL2 OR CCL3 OR CCL4 OR CCL5 OR CCL6 OR CCL7 OR CCL8 OR CCL9 OR CCL10 OR CCL11 OR CCL12 OR CCL13 OR CCL14 OR CCL15 OR CCL16 OR CCL17 OR CCL18 OR CCL19 OR CCL20 OR CCL21 OR CCL22 OR CCL23 OR CCL24 OR CCL25 OR CCL26 OR CCL27 OR CCL28 OR CXCL1 OR CXCL2 OR CXCL3 OR CXCL4 OR CXCL5 OR CXCL6 OR CXCL7 OR CXCL8 OR CXCL9 OR CXCL10 OR CXCL11 OR CXCL12 OR CXCL13 OR CXCL14 OR CXCL15 OR CXCL16 OR CXCL17 OR XCL1 OR XCL2 OR CX3CL1 OR tnf* OR “tumour necrosis factor*” OR “tumor necrosis factor*” OR interferon* OR IFN*) OR SU=(cytokine* OR interleukin* OR chemokine* OR il OR il1 OR il1a OR il1b OR il2 OR il3 OR il4 OR il5 OR il6 OR il7 OR il8 OR il9 OR il10 OR il11 OR il12 OR il13 OR il14 OR il15 OR il16 OR il17 OR il18 OR il19 OR il20 OR il21 OR il22 OR il23 OR il24 OR il25 OR il26 OR il27 OR il28 OR il28* OR il29 OR il30 OR il31 OR il32 OR il33 OR il35 OR il36 OR CCL1 OR CCL2 OR CCL3 OR CCL4 OR CCL5 OR CCL6 OR CCL7 OR CCL8 OR CCL9 OR CCL10 OR CCL11 OR CCL12 OR CCL13 OR CCL14 OR CCL15 OR CCL16 OR CCL17 OR CCL18 OR CCL19 OR CCL20 OR CCL21 OR CCL22 OR CCL23 OR CCL24 OR CCL25 OR CCL26 OR CCL27 OR CCL28 OR CXCL1 OR CXCL2 OR CXCL3 OR CXCL4 OR CXCL5 OR CXCL6 OR CXCL7 OR CXCL8 OR CXCL9 OR CXCL10 OR CXCL11 OR CXCL12 OR CXCL13 OR CXCL14 OR CXCL15 OR CXCL16 OR CXCL17 OR XCL1 OR XCL2 OR CX3CL1 OR tnf* OR “tumour necrosis factor*” OR “tumor necrosis factor*” OR interferon* OR IFN*) OR WC=(cytokine* OR interleukin* OR chemokine* OR il OR il1 OR il1a OR il1b OR il2 OR il3 OR il4 OR il5 OR il6 OR il7 OR il8 OR il9 OR il10 OR il11 OR il12 OR il13 OR il14 OR il15 OR il16 OR il17 OR il18 OR il19 OR il20 OR il21 OR il22 OR il23 OR il24 OR il25 OR il26 OR il27 OR il28 OR il28* OR il29 OR il30 OR il31 OR il32 OR il33 OR il35 OR il36 OR CCL1 OR CCL2 OR CCL3 OR CCL4 OR CCL5 OR CCL6 OR CCL7 OR CCL8 OR CCL9 OR CCL10 OR CCL11 OR CCL12 OR CCL13 OR CCL14 OR CCL15 OR CCL16 OR CCL17 OR CCL18 OR CCL19 OR CCL20 OR CCL21 OR CCL22 OR CCL23 OR CCL24 OR CCL25 OR CCL26 OR CCL27 OR CCL28 OR CXCL1 OR CXCL2 OR CXCL3 OR CXCL4 OR CXCL5 OR CXCL6 OR CXCL7 OR CXCL8 OR CXCL9 OR CXCL10 OR CXCL11 OR CXCL12 OR CXCL13 OR CXCL14 OR CXCL15 OR CXCL16 OR CXCL17 OR XCL1 OR XCL2 OR CX3CL1 OR tnf* OR “tumour necrosis factor*” OR “tumor necrosis factor*” OR interferon* OR IFN*) ) AND (TS=(human* OR man OR men OR woman OR women OR person* OR people OR individual* OR patient* OR child* OR adolescent* OR newborn* OR toddler* OR senior*) OR TI=(human* OR man OR men OR woman OR women OR person* OR people OR individual* OR patient* OR child* OR adolescent* OR newborn* OR toddler* OR senior*) OR AB=(human* OR man OR men OR woman OR women OR person* OR people OR individual* OR patient* OR child* OR adolescent* OR newborn* OR toddler* OR senior*) OR AK=(human* OR man OR men OR woman OR women OR person* OR people OR individual* OR patient* OR child* OR adolescent* OR newborn* OR toddler* OR senior*) OR KP=(human* OR man OR men OR woman OR women OR person* OR people OR individual* OR patient* OR child* OR adolescent* OR newborn* OR toddler* OR senior*) OR SU=(human* OR man OR men OR woman OR women OR person* OR people OR individual* OR patient* OR child* OR adolescent* OR newborn* OR toddler* OR senior*) OR WC=(human* OR man OR men OR woman OR women OR person* OR people OR individual* OR patient* OR child* OR adolescent* OR newborn* OR toddler* OR senior*))
