# Supplementary material for: How do serum cytokine levels change in myocarditis and inflammatory dilated cardiomyopathy relative to healthy individuals? A protocol for a systematic review and meta-analysis
Source: BMJ Open. 2025 Feb 7;15(2):e087052. doi: 10.1136/bmjopen-2024-087052 (PMC11808896; doi:10.1136/bmjopen-2024-087052)
Supplement: Supplementary file 3 [file bmjopen-2024-087052.reviewer_comments.pdf]

PEER REVIEW HISTORY

BMJ Open publishes all reviews undertaken for accepted manuscripts. Reviewers are asked to complete a checklist review form and are provided with free text boxes to elaborate on their assessment. These free text comments are reproduced below.

ARTICLE DETAILS

Title (Provisional)

How do serum cytokine levels change in myocarditis and inflammatory dilated cardiomyopathy relative to healthy individuals? A protocol for a systematic review and meta-analysis

Authors

Lewandowski, Piotr; Baron, Maciej; Rojek, Marcin A.; Goławski, Marcin; Krystek, Karol; Żegleń, Magdalena; Zytkiewicz, Pascal; Kancerek, Jakub Kancerek; Lis, Klaudia Gabriela; Warecki, Jakub; Kufel, Jakub; Kuśpiel, Kamila; Wojnicz, Romuald

---

VERSION 1 - REVIEW

**Reviewer**	**1**
**Name**	**Li, Jinrong**
**Affiliation**	**Sichuan University, Department of Pediatrics**
**Date**	**12-Jun-2024**
**COI**	**NO**

This article is about Cytokines serum levels in mycocardis and inflammatory

A systematic evaluation of the research plan for dilated cardiopathy. The research protocol does not involve any results or conclusions, and is an article about the research protocol. The main research of the article is divided into three directions:

This will be the first attempt to summarize the studies on cytokine levels in

myocarditis.

This study will follow PRISMA-P guidelines, risk of bias and certainty of evidence

assessment and will utilize meta-analysis approach to provide high quality results.

The main limitation of this study could be a high heterogeneity of included studies and

patient cohorts, and this may lead to inconsistent results

I hope to add two research topics:

However, based on the prediction of clinical prognosis, it is recommended to increase the difference in cytokine profiles between acute and chronic myocarditis

The relationship between cytokines and the prognosis of myocarditis.

**Reviewer**	**2**
**Name**	**Rubiś, Paweł**
**Affiliation**	**Jagiellonian University**
**Date**	**17-Jul-2024**
**COI**	**Nothing to declecre**

The present manuscript, written by Lewandowski Piotr and colleagues, is a description of the protocol designed to perform systematic review and meta-analysis on circulating cytokines in myocarditis and inflammatory dilated cardiomyopathy. The design and merit of the protocol is presented in a great details, and the subject is highly relevant for the poorly explored field of the role (if any) of cytokines in myocarditis and dilated cardiomyopathy. The study design is well-thought and it seems that there is not really much to add to it. Moreover, the authors pledged to strictly follow widely-approved PRIMSA, which guarantees high-quality research. Overall, the article is very well written and easily understandable.

**Reviewer**	**3**
**Name**	**Harding, Daniel**
**Affiliation**	**Queen Mary University of London William Harvey Research Institute**
**Date**	**15-Oct-2024**
**COI**	**None**

This review protocol is adequate - I have no concerns.

VERSION 1 - AUTHOR RESPONSE

Response to Reviewer 1:

Reviewer: 1
Dr. Jinrong Li, Sichuan University
Comments to the Author:
This article is about Cytokines serum levels in mycocardis and inflammatory
A systematic evaluation of the research plan for dilated cardiopathy. The research protocol does not involve any results or conclusions, and is an article about the research protocol. The main research of the article is divided into three directions:

This will be the first attempt to summarize the studies on cytokine levels in myocarditis.
This study will follow PRISMA-P guidelines, risk of bias and certainty of evidence assessment and will utilize meta-analysis approach to provide high quality results.
The main limitation of this study could be a high heterogeneity of included studies and patient cohorts, and this may lead to inconsistent results
I hope to add two research topics:
However, based on the prediction of clinical prognosis, it is recommended to increase the difference in cytokine profiles between acute and chronic myocarditis
The relationship between cytokines and the prognosis of myocarditis.

Dear Dr. Jinrong Li,

We thank you for your thorough review and thoughtful suggestions, which we found to be very insightful and valuable.

In response to your suggestions, we have revised the manuscript to include two additional study objectives, as follows:

1. Do the serum/plasma concentrations in chronic myocarditis and inflammatory dilated cardiomyopathy patients differ compared to acute myocarditis patients?
2. What is the relationship between cytokine concentrations and the prognosis of myocarditis?

We have also updated the scope of data extraction in our methodology to ensure these objectives are appropriately addressed.

We believe that these enhancements will further improve the scope and relevance of the protocol. Once again, we thank you for your constructive input, which has significantly contributed to refining our work.

With kind regards,
Piotr Lewandowski and colleagues

Response to Reviewer 2:

Reviewer: 2
Prof. Paweł Rubiś, Jagiellonian University, Krakow Specialist Hospital named after John Paul II
Comments to the Author:
The present manuscript, written by Lewandowski Piotr and colleagues, is a description of the protocol designed to perform systematic review and meta-analysis on circulating cytokines in myocarditis and inflammatory dilated cardiomyopathy. The design and merit of the protocol is presented in a great details, and the subject is highly relevant for the poorly explored field of the role (if any) of cytokines in myocarditis and dilated cardiomyopathy. The study design is well-thought and it seems that there is not really much to add to it. Moreover, the authors pledged to strictly follow widely-approved PRIMSA, which guarantees high-quality research. Overall, the article is very well written and easily understandable.

Dear Prof. Paweł Rubiś,

We sincerely thank you for taking the time to review our manuscript and for your positive and encouraging feedback. We appreciate your recognition of the relevance of

our study design and the systematic approach we have outlined.

Your encouraging feedback about the article being "well-written and easy to understand" inspires us to uphold this standard throughout the study and in future publications. Thank you once again for your thoughtful review and support for our work.

With kind regards,
Piotr Lewandowski and colleagues

Response to Reviewer 3:
Reviewer: 3
Dr. Daniel Harding, Queen Mary University of London William Harvey Research Institute
Comments to the Author:
This review protocol is adequate - I have no concerns.

Dear Dr. Daniel Harding,

Thank you for your review and for your concise and supportive feedback. We are pleased that you found our review protocol adequate and that you have no concerns regarding our work.

Your positive assessment is greatly appreciated and motivates us to proceed confidently with the study.

With best regards,
Piotr Lewandowski and colleagues
